# Longitudinal changes in laboratory parameters and QTc during isavuconazole therapy in Japanese patients with hematologic malignancies

**DOI:** 10.1371/journal.pone.0354816

**Published:** 2026-07-29

**Authors:** Koichi Ohata, Ryo Kobayashi, Daichi Watanabe, Yuto Yamada, Takashi Niwa, Mio Wada, Momoko Ohashi, Yuto Kaneda, Yoshikazu Ikoma, Takuro Matsumoto, Nobuhiko Nakamura, Hiroshi Nakamura, Nobuhiro Kanemura, Masahito Shimizu, Akio Suzuki

**Affiliations:** 1 Department of Pharmacy, Gifu University Hospital, Gifu, Japan; 2 Laboratory of Advanced Medical Pharmacy, Gifu Pharmaceutical University, Gifu, Japan; 3 Medical Affairs Department, Asahi Kasei Therapeutics Corporation, Chiyoda-ku, Tokyo, Japan; 4 Department of Hematology and Infectious Disease, Gifu University Hospital, Gifu, Japan; 5 Center for Nutrition Support and Infection Control, Gifu University Hospital, Gifu, Japan; Gulu University, UGANDA

## Abstract

**Background:**

Isavuconazole (ISCZ) is an azole antifungal with a low risk of QTc prolongation and no requirement for renal dose adjustment. However, real-world data in Asian patients remain limited.

**Methods:**

We retrospectively evaluated 45 patients who received ISCZ between April 2023 and March 2025. Laboratory parameters and QTc values (QTcB/QTcF) were extracted from medical records. Longitudinal changes were assessed using linear mixed-effects models. In patients receiving arsenic trioxide (ATO), QTc trajectories were compared among ATO alone, ATO with ISCZ (ATO-ISCZ), and ATO with other antifungals.

**Results:**

Grade ≥2 elevations in AST and ALT occurred in 11.1% and 13.3% of patients, respectively, and were transient. No significant deterioration in laboratory parameters was observed. In the ATO subgroup, QTc values in the ATO-ISCZ group were lower than those in the ATO group from Day 30, with the largest difference at Day 70 (−45.5 ms for QTcB and −42.3 ms for QTcF; both p < 0.001). The ATO–other antifungals group showed similar trends to the ATO group. No clinically significant cardiac events were observed.

**Conclusions:**

ISCZ showed a favorable safety profile in Japanese patients with hematologic malignancies and may be a safe option during QT-prolonging therapies.

## Introduction

Invasive fungal infections (IFIs) are serious complications associated with high mortality in patients with hematologic malignancies who undergo intensive chemotherapy or immunosuppressive therapy, particularly those with prolonged neutropenia or graft-versus-host disease (GVHD) after allogeneic hematopoietic stem cell transplantation (HSCT) [1,2]. Therefore, appropriate prophylaxis and early treatment of IFIs are crucial for improving clinical outcomes in this population [3].

Isavuconazole is a broad‑spectrum triazole antifungal agent whose prodrug, isavuconazonium sulfate, is approved for the treatment of invasive aspergillosis and mucormycosis [4,5]. Hereafter, we refer to both forms collectively as ISCZ. ISCZ has several clinically favorable properties: it is available in both intravenous and oral formulations, and its high oral bioavailability facilitates switching from intravenous to oral therapy [6,7]. In addition, ISCZ does not generally require therapeutic drug monitoring (TDM) [8,9], in clear contrast to voriconazole (VRCZ), for which TDM is recommended due to its nonlinear pharmacokinetics [10,11]. ISCZ is not primarily eliminated via the renal route, and dose adjustment is not required in patients with renal impairment. Moreover, it appears to carry a lower risk of drug–drug interactions than other azoles [12,13].

Another important characteristic of ISCZ is its absence of a clinically relevant risk of QTc interval prolongation, a common concern with VRCZ and other azoles. In a thorough QT study, ISCZ did not demonstrate concentration-dependent QTc prolongation [14]. In fact, pooled analyses of clinical trial datasets and real-world evidence have shown that ISCZ is associated with QTc shortening rather than prolongation [14,15]. Based on this unique profile, it has been suggested that ISCZ can be used safely even in patients with pre-existing QTc prolongation [16]. In contrast, several antineoplastic agents frequently used in the treatment of hematologic malignancies, such as arsenic trioxide (ATO), are known to prolong the QT interval and increase the risk of ventricular arrhythmias [17–19]. Accordingly, ISCZ may offer a clinically meaningful advantage from a cardiovascular safety standpoint on co-administration with QT-prolonging agents.

However, real-world data on ISCZ use in Asian populations, including Japanese patients, remain limited. Most available evidence comes from international phase 3 trials [4,5] and a specific Japanese phase 3 study [20]. Although retrospective real-world reports in Asian patients with hematologic diseases have begun to emerge [21,22], these studies are restricted by relatively small sample sizes and specific patient populations. In particular, data on the impact of ISCZ on QTc interval in patients receiving ATO-containing regimens are markedly limited.

The present study aimed to retrospectively evaluate the clinical use and safety of ISCZ in patients treated at the Department of Hematology and Infectious Diseases, Gifu University Hospital. We specifically focused on longitudinal changes in laboratory parameters reflecting hepatic and renal function, as well as QTc interval dynamics. In addition, we also explored QTc changes in patients receiving high-risk ATO-containing regimens with and without concomitant ISCZ.

## Methods

### Patients

This retrospective study included 45 consecutive patients who received ISCZ at the Department of Hematology and Infectious Diseases, Gifu University Hospital, between April 1, 2023, and March 31, 2025. Data were obtained from electronic medical records. Patients aged <18 years and those in whom ISCZ was discontinued within 3 days of initiation, making safety evaluation difficult, were excluded. The primary objective of this study was to evaluate the clinical use and safety of ISCZ in real-world practice, with a focus on longitudinal changes in laboratory parameters. Additionally, to investigate the impact of ISCZ on drug-induced QTc prolongation, we exploratorily assessed QTc trajectories in patients receiving high-risk ATO-containing regimens by comparing those with and without concomitant ISCZ.

### Data collection

The following data were collected: patient demographics (age, sex), underlying hematologic malignancy, history of HSCT, Eastern Cooperative Oncology Group Performance Status (ECOG PS), route of administration, and duration of ISCZ therapy. Laboratory parameters collected during ISCZ therapy included aspartate aminotransferase (AST), alanine aminotransferase (ALT), total bilirubin (T-Bil), γ-glutamyl transferase (γ-GTP), alkaline phosphatase (ALP), lactate dehydrogenase (LDH), serum creatinine (Cre), neutrophil count (Neut), white blood cell count (WBC), platelet count (PLT), hemoglobin (HGB), sodium (Na), potassium (K), and chloride (CL). Electrocardiogram (ECG) findings, specifically QTc intervals, were also collected. Laboratory abnormalities were graded according to the Common Terminology Criteria for Adverse Events (CTCAE), version 5.0. Data used in the final analysis were extracted from the electronic medical records for research purposes between February 4, 2025, and March 19,2026.

### Assessment of QTc interval

**Fig 1:**
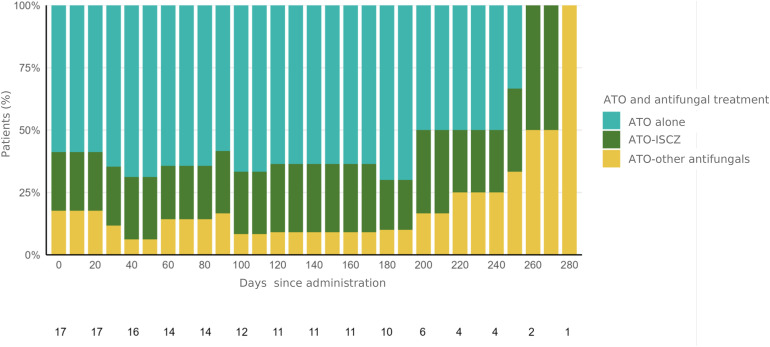
Longitudinal distribution of treatment status. Stacked bar chart showing the proportions of the three treatment groups (ATO alone, ATO-ISCZ, and ATO-other antifungals) among 17 patients over the course of arsenic trioxide therapy. The numbers below the x-axis indicate the total number of patients e.valuated at each time point. Abbreviations: ATO, arsenic trioxide; ISCZ, isavuconazole.

QTc intervals were obtained from standard 12-lead ECGs recorded using hospital ECG systems, and automated QTc values calculated by the device’s built-in algorithm were used for analysis. Baseline QTc was defined as the ECG measurement performed at the start of an ATO-containing regimen. During treatment, all ECGs performed at the discretion of the treating physician were reviewed, and QTc values recorded during periods of ATO administration were included in the analysis. To assess the impact of different antifungal strategies on QTc dynamics during high-risk therapy, we evaluated 17 patients who received ATO-containing regimens, including the 4 patients in the ISCZ treatment cohort (n = 45) who received concomitant ATO. Because the status of concomitant antifungal therapy could change over time within an individual patient, each observation point was categorized into one of the following three groups based on the therapy administered at the time of the ECG, namely (1) ATO alone; (2) ATO with concomitant ISCZ (ATO-ISCZ); or (3) ATO with other concomitant antifungals, including itraconazole, fluconazole, voriconazole, or micafungin with or without liposomal amphotericin B (ATO-other antifungals). The longitudinal distribution and transitions of these treatment statuses are illustrated in Fig 1. All available QTc values recorded during periods of ATO administration were included in the linear mixed-effects model analysis, which appropriately accounted for these time-varying covariates.

### Statistical analysis

Continuous variables are presented as the median (range) or median (interquartile range), and categorical variables as numbers (%). Longitudinal changes in laboratory parameters during ISCZ therapy, as well as QTc intervals among the three groups defined in Section 2.3, were evaluated using linear mixed-effects models, accounting for repeated measurements within individuals. Random-intercept models were applied, with individual patients treated as random effects. For descriptive visualization of QTc dynamics, changes from baseline were calculated for each patient, and individual trajectories were visualized as spaghetti plots against days since treatment initiation. QTc analyses were performed using the observed QTc values (QTcB and QTcF) as outcome variables. To account for potential non-linear trajectories in patients receiving ATO-containing regimens, time (days) was modeled using a restricted cubic spline with four knots in a linear mixed-effects model. In these models, treatment group, time (modeled as a spline), and their interaction were included as fixed effects, with baseline QTc and ATO exposure (yes/no) at each measurement occasion included as a covariate for adjustment. Group-specific trajectories were visualized based on the predicted values from these non-linear models. Furthermore, to quantify the differences between groups at specific time points, estimated marginal means were calculated, and pairwise comparisons between the ATO group and other two groups were performed at 10-day intervals (from day 10–100) using Tukey adjustment within each time point. A two-sided p value of < 0.05 was considered statistically significant. Statistical analyses were performed using EZR (Saitama Medical Center, Jichi Medical University) [23] and R (version 4.5.1; The R Foundation for Statistical Computing) [24].

### Ethical considerations

This study was approved by the Ethics Committee of the Graduate School of Medicine, Gifu University (approval number: 2024−272). Owing to the retrospective observational design using existing medical records, the requirement for written informed consent was waived by the ethics committee. Information regarding the study was made publicly available, and patients were given the opportunity to opt out in accordance with institutional policies. The data were handled in a de-identified manner, and analyses were conducted after removal of directly identifiable personal information.

## Results

### Patient characteristics

A total of 45 patients who received ISCZ during the study period were included in the analysis. Underlying hematologic malignancies included acute myeloid leukemia, acute lymphoblastic leukemia, non-Hodgkin lymphoma, and myelodysplastic syndrome, among others (Table 1). Among patients who underwent hematopoietic stem cell transplantation, the largest group consisted of patients who received allogeneic hematopoietic stem cell transplantation (HSCT). ISCZ was used for the treatment of both documented or suspected IFI and for prophylaxis in high-risk patients. All patients received oral ISCZ.

**Table 1:** Patient characteristics.

Characteristic	Value
Age, years, median (range)	66.0 (21–85)
Male/Female, n (%)	26/19 (57.8/42.2)
Body mass index, kg/m², median (range)	21.5 (14.6–30.1)
Duration of ISCZ administration, days, median (range)	130 (6–339)
Primary disease, n (%)	
Non-Hodgkin lymphoma (NHL)	19 (42.2)
Acute myeloid leukemia (AML)	14 (31.1)
Myelodysplastic syndromes (MDS)	8 (17.8)
Acute lymphoblastic leukemia (ALL)	2 (4.4)
Chronic lymphocytic leukemia (CLL)	1 (2.2)
Multiple myeloma (MM)	1 (2.2)
Hematopoietic stem cell transplantation, n (%)	
Autologous	1 (2.2)
Allogeneic	16 (35.6)
None	28 (62.2)
Acute GVHD, n (%)	
Skin involvement	3 (6.7)
Gastrointestinal involvement	8 (17.8)
GVHD grade, n (%)	
Grade 1/2	1 (2.2)/10 (22.2)
Prior infections within 3 months, n (%)	
Deep-seated mycosis	8 (17.8)
Viral infection	4 (8.9)
Bacterial infection	6 (13.3)
None	27 (60.0)
Baseline laboratory values, median (range)	
AST, U/L	21.0 (11–207)
ALT, U/L	22.0 (8–389)
T-bil, mg/dL	0.7 (0.3–3.2)
ALB, g/dL	3.7 (1.8–4.6)
Cre, mg/dL	0.78 (0.40–1.67)
WBC, /µL	4,150 (170–52,070)
Neut, /µL	2,360 (4–26,530)
Hb, g/dL	9.2 (6.2–15.4)
PLT, × 10⁴/µL	14.1 (1.6–265.0)
Na, mEq/L	139 (128–145)
K, mEq/L	4.3 (3.5–5.6)
CL, mEq/L	105 (96–109)

### Longitudinal changes in laboratory parameters

**Fig 2:**
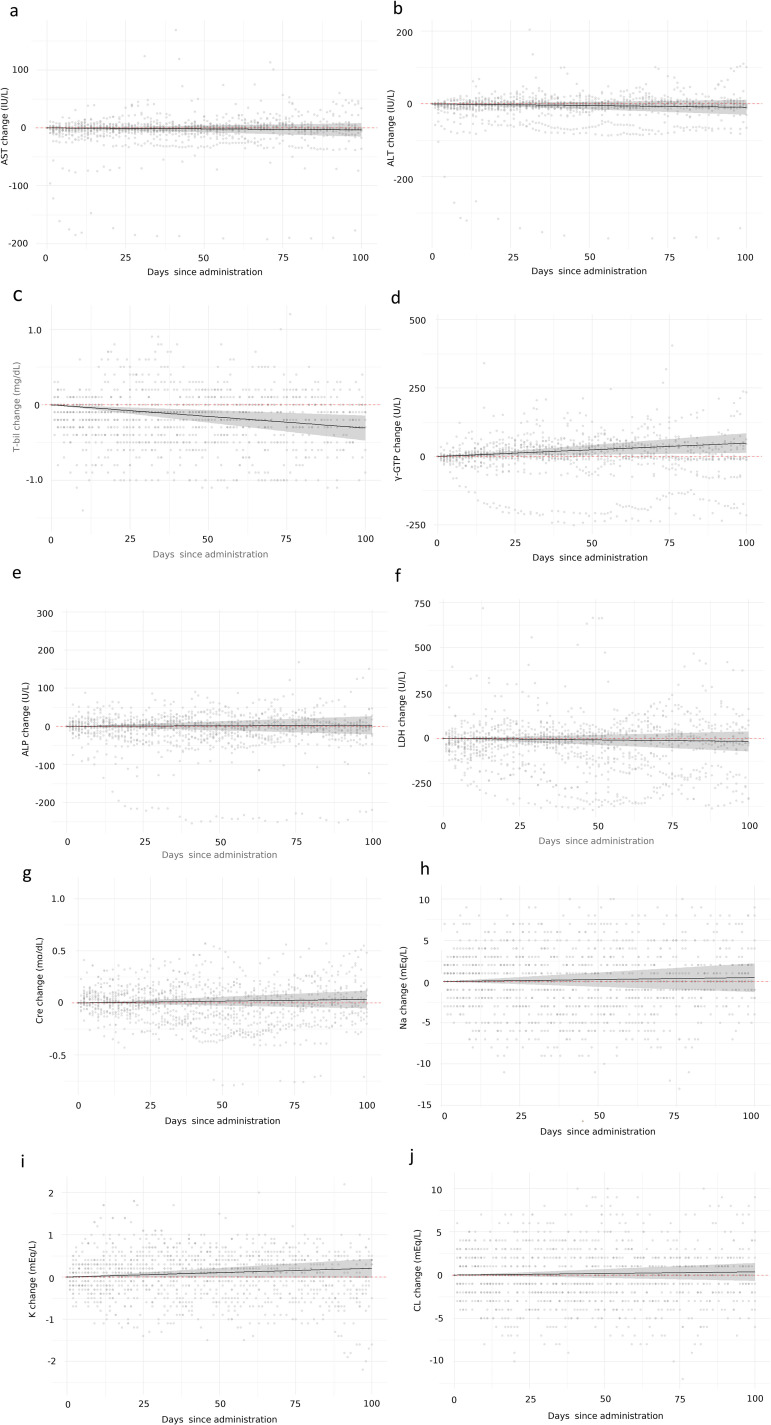
Longitudinal trajectories of clinical laboratory parameters during isavuconazole therapy. The solid lines represent the estimated marginal means derived from linear mixed-effects models, with shaded areas indicating 95% confidence intervals. Linear mixed-eTheffects modeling revealed no evidence of a unidirectional and clinically meaningful deterioration over time in any of the evaluated parameters, including hepatic markers (AST, ALT, T-Bil, γ-GTP, ALP, LDH), renal function (Cre), or electrolytes (Na, K, CL). The dashed red lines represent the baseline value (zero change), and scatter plots show individual data points for the 45 patients.

During ISCZ therapy, CTCAE grade ≥ 2 elevations in liver enzymes were observed in 11.1% (5/45) patients for AST and 13.3% (6/45) for ALT. These abnormalities were transient in all cases, and ISCZ could be continued in all cases without dose modification. Linear mixed-effects modeling revealed no evidence of a unidirectional and clinically meaningful deterioration over time in any of the evaluated parameters, including hepatic markers (AST, ALT, T-Bil, γ-GTP, ALP, LDH), renal function (Cre), or electrolytes (Na, K, CL) (Fig 2). Even among patients who already had abnormal hepatic or renal function at the start of ISCZ therapy, follow-up values were generally similar to or lower than baseline values, and no clear signal suggesting ISCZ-associated worsening of hepatic or renal function was seen.

### QTc interval dynamics

**Fig 3:**
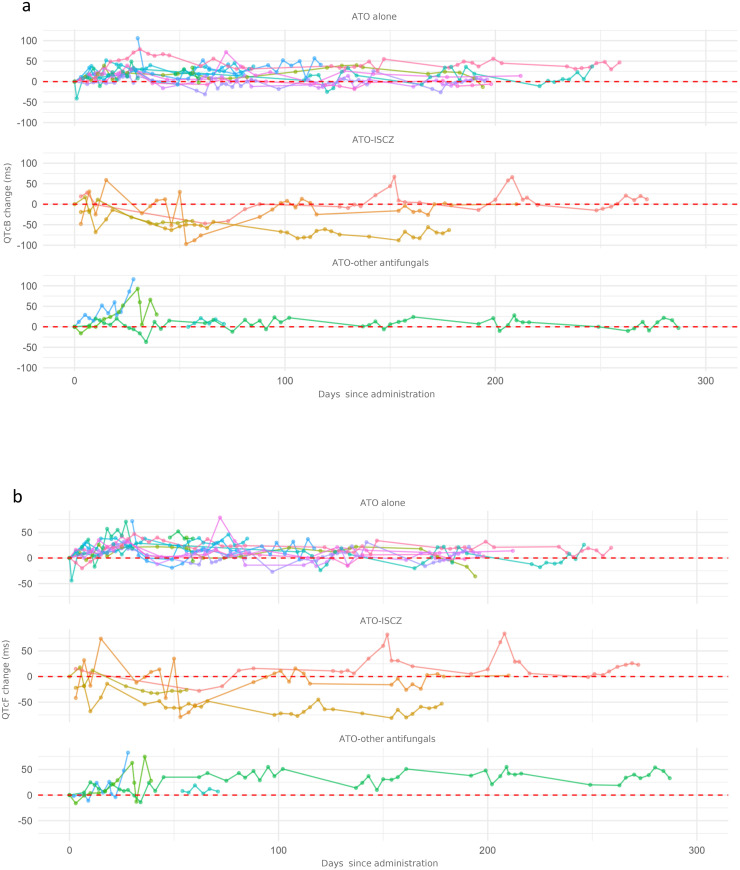
Individual longitudinal changes in QTcB and QTcF intervals under ATO-containing regimens. Longitudinal changes from baseline in (a) QTcB and (b) QTcF intervals are shown for individual patients, stratified by treatment group: ATO, ATO-ISCZ and ATO-other antifungals. Each line represents an individual patient’s trajectory. The red dashed lines indicate baseline (zero change).

**Fig 4:**
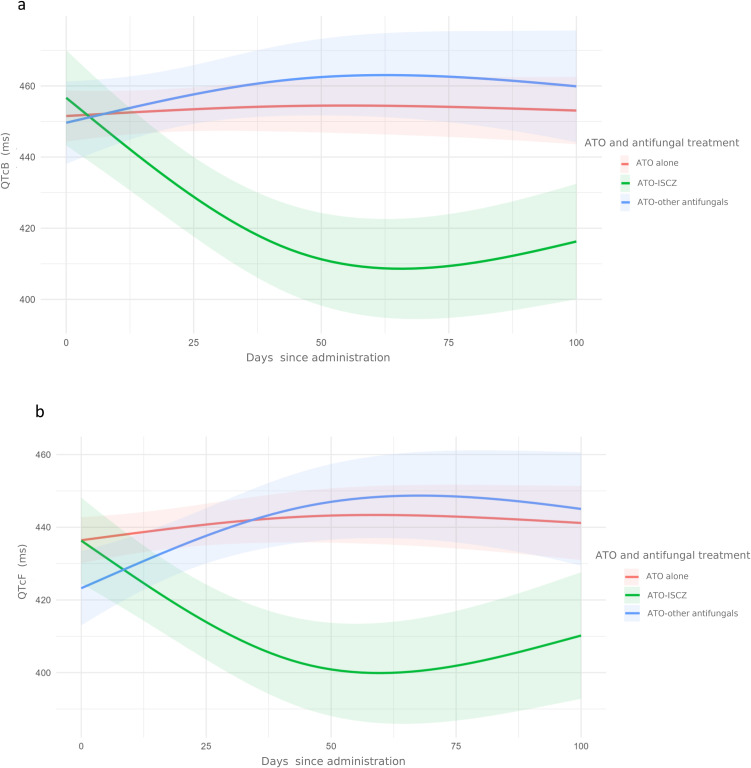
Predicted trajectories of QTcB and QTcF intervals based on non-linear mixed-effects modeling. Estimated marginal means for (a) absolute QTcB values and (b) absolute QTcF values over time, modeled using restricted cubic splines with four knots to account for non-linear dynamics. Predicted values are adjusted for baseline levels. Patients are stratified into three treatment groups: ATO alone (red lines), ATO-ISCZ (green lines), and ATO-other antifungal agents (blue lines). Shaded areas represent 95% confidence intervals.

Individual longitudinal changes in QTcB and QTcF are presented as spaghetti plots in Fig 3. In the ATO alone group and ATO-other antifungals group, a tendency toward QTc prolongation from baseline was observed in many patients. In contrast, in the ATO-ISCZ group, while some patients exhibited transient increases in QTc intervals, many trajectories remained stable or demonstrated negative changes (shortening) throughout the observation period. These observations were quantified using a multivariable linear mixed-effects model with restricted cubic splines (Fig 4). Quantitative comparisons using estimated marginal means are shown in Table 2. Analysis revealed that the ATO–ISCZ group exhibited significantly lower QTc values compared with the ATO group starting from Day 30 (QTcB difference: −29.6 ms, 95% CI [−47.4 to −11.9], p < 0.001; QTcF difference: −31.2 ms, 95% CI [−47.3 to −15.1], p < 0.001). The shortening effect was most pronounced at Day 70, with an estimated difference of −45.5 ms for QTcB (95% CI [−67.2 to −23.8], p < 0.001) and −42.3 ms for QTcF (95% CI [−64.2 to −20.4], p < 0.001). Furthermore, significantly shorter intervals in the ATO-ISCZ group were maintained at Day 90 (QTcB difference: −40.6 ms, p < 0.001; QTcF difference: −35.4 ms, p = 0.004).

**Table 2:** Estimated differences in QTc intervals relative to the ATO group.

Day	Comparison (vs. ATO)	QTcB Diff (ms)	95% CI	p-value	QTcF Diff (ms)	95% CI	p-value
10	ATO-ISCZ	-7.3	[-25.0, 10.4]	0.581	-11.3	[-26.6, 4.0]	0.183
	ATO-other antifungals	+0.7	[-11.7, 13.0]	0.991	-9.0	[-19.7, 1.7]	0.116
30	ATO-ISCZ	-29.6	[-47.4, -11.9]	<0.001	-31.2	[-47.3, -15.1]	<0.001
	ATO-other antifungals	+5.2	[-4.8, 15.2]	0.436	-1.3	[-10.3, 7.7]	0.933
50	ATO-ISCZ	-43.2	[-63.1, -23.2]	<0.001	-42.3	[-61.5, -23.1]	<0.001
	ATO-other antifungals	+8.0	[-4.3, 20.4]	0.278	+3.8	[-7.8, 15.3]	0.721
70	ATO-ISCZ	-45.5	[-67.2, -23.8]	<0.001	-42.3	[-64.2, -20.4]	<0.001
	ATO-other antifungals	+8.6	[-5.3, 22.5]	0.313	+5.5	[-7.7, 18.8]	0.587
90	ATO-ISCZ	-40.6	[-64.0, -17.1]	<0.001	-35.4	[-60.0, -10.8]	0.004
	ATO-other antifungals	+7.6	[-8.9, 24.2]	0.514	+4.8	[-11.0, 20.6]	0.747

ATO: Arsenic trioxide, ATO-ISCZ: ATO with co-administration of ISCZ, ATO-other antifungals: ATO with co-administration of other antifungals

In contrast, the ATO–other antifungals group showed no significant difference from the arsenic trioxide alone group at any time point, with trajectories often trending toward prolongation.

Additionally, the ATO treatment groups showed no clinically significant cardiac events, including torsades de pointes, symptomatic ventricular arrhythmias, or sudden cardiac death, regardless of the type of concomitant antifungal therapy.

## Discussion

In this study, we found that QTc intervals remained at relatively shorter levels in the ISCZ co-administration group compared to the arsenic trioxide (ATO) alone group, even under exposure to ATO, which carries a high risk of QT prolongation. We also found that liver function abnormalities during ISCZ therapy were infrequent and transient, with CTCAE grade ≥2 elevations in AST occurring in 11.1% (5/45) and ALT in 13.3% (6/45) of patients, and ISCZ could be continued in all cases without dose modification. These findings are particularly valuable because real-world data on ISCZ use in Asian populations, including Japanese patients, remain limited [20–22], and the present study provides important evidence regarding the safety of ISCZ in clinical scenarios with a high risk of QT prolongation.

In the multivariable analysis using a non-linear model, the significant negative differences observed in the ATO–ISCZ group (with estimated differences reaching approximately −30 to −45 ms compared to the ATO alone group; Table 2) likely reflect ISCZ’s unique pharmacological property of QTc shortening [14,15]. This finding indicates that the QTc-shortening effect of ISCZ may contribute to the maintenance of stable QTc intervals even during concomitant therapy with ATO, an agent known for its potent prolonging effects. Furthermore, this effect did not deteriorate over time: the absence of a significant interaction between days since initiation and the treatment group indicates that the addition of ISCZ to ATO therapy did not lead to further QTc prolongation or its exacerbation throughout the observation period. These results suggest that the QTc-modulating effect of ISCZ is sustained during extended co-administration with ATO.

Although the estimated QTc differences were numerically substantial, their clinical implications should be interpreted cautiously. In routine clinical practice, QTc-related risk is assessed comprehensively on the basis of absolute QTc values, changes from baseline, concomitant risk factors, and the occurrence of arrhythmias, rather than QTc shortening itself. Therefore, the present findings should not be interpreted as evidence that QTc shortening per se represents a therapeutic benefit or that ISCZ prevents clinically significant arrhythmias.

Rather, the lower QTc values observed in the ATO-ISCZ group may suggest that concomitant ISCZ did not exacerbate ATO-associated QTc prolongation and may have contributed to maintaining lower QTc values during ATO therapy. However, because no clinically significant cardiac events occurred and this study was not powered to evaluate such outcomes, the effect of these QTc differences on arrhythmia risk remains uncertain.

Typically, the concomitant use of other azoles (e.g., voriconazole or itraconazole) during ATO therapy necessitates intensive monitoring or avoidance due to the risk of additive QT prolongation [17–19]. In contrast, the ATO–ISCZ group in our study showed an overall stable QTc trajectory, while the ATO–other antifungals group showed a trajectory similar to that of the ATO alone group. This finding suggests that ISCZ is an appropriate option from a cardiovascular safety perspective when administering ATO-containing regimens [25], and distinguishes ISCZ from other azole antifungals in this high-risk clinical setting.

The rates of hepatic enzyme elevation observed in our study are not higher than the reported incidence of VRCZ-related hepatotoxicity (up to 16%) [26] or other azole antifungals. Moreover, systematic reviews [27], large-scale registry data [28], and comparative studies with VRCZ [29,30] have consistently suggested that ISCZ may have a lower risk of hepatotoxicity and nephrotoxicity. Our results support this favorable safety profile. Beyond hepatic and cardiovascular safety, ISCZ offers practical advantages: it does not require therapeutic drug monitoring [8,9,27], has relatively stable pharmacokinetics with no dose adjustment for renal impairment [6,12], and has a less potent inhibitory effect on CYP enzymes compared to other azoles [13,31]. The lack of a TDM requirement is particularly relevant in Japanese and other Asian populations, where the prevalence of CYP2C19 poor metabolizers is higher than in other ethnicities [32], making dose optimization of CYP2C19-dependent azoles such as VRCZ more challenging [9,27]. These combined characteristics make ISCZ a convenient and safe option for managing invasive fungal infections in hematopoietic stem cell transplant recipients who require multiple concomitant medications.

There are several limitations to this study. First, this was a retrospective, single-center study with a small overall sample size. In particular, the QTc analysis included only 17 patients who received ATO-containing regimens, and the ATO-ISCZ group comprised a limited number of patients. Therefore, the estimated between-group differences in QTc should be interpreted cautiously, as they may be sensitive to patient-level characteristics, treatment selection, and the distribution of repeated ECG measurements. Although the use of linear and non-linear mixed-effects models allowed us to incorporate all available longitudinal observations and partially improve statistical efficiency, these approaches cannot fully overcome the limited number of patients or establish a causal effect of ISCZ on QTc dynamics.

Second, because of the retrospective design, ECG timing was determined by clinical judgment rather than by a standardized schedule. Consequently, the frequency and timing of ECG assessments may have differed according to the patient’s clinical condition, treatment phase, or perceived cardiovascular risk, potentially introducing surveillance bias and limiting direct comparability of QTc trajectories across groups. Nevertheless, this real-world measurement approach reflects actual clinical practice and may enhance the external relevance of our findings. Prospective multicenter studies with larger ATO-treated cohorts and prespecified ECG assessment schedules are needed to confirm these observations.

In summary, ISCZ demonstrated a favorable safety profile in Japanese patients with hematologic malignancies, with no clinically significant deterioration in laboratory parameters and maintenance of shorter QTc intervals even during co-administration with ATO. These results suggest that ISCZ’s characteristics, including no requirement for TDM or renal dose adjustment, a lower risk of QTc prolongation, and fewer drug–drug interactions, are generally reproducible in this population. Our findings support ISCZ as an important candidate for antifungal management in patients with hematologic malignancies, combining both safety and clinical convenience. Future multicenter, prospective studies with larger sample sizes and standardized ECG scheduling are warranted to validate these findings and to further quantify the QTc-modulating effect of ISCZ during ATO-containing regimens.

## Supporting information

S1 Data Minimal de-identified dataset underlying the findings of this study. (XLSX)
