# Supplementary material for: Longitudinal changes in laboratory parameters and QTc during isavuconazole therapy in Japanese patients with hematologic malignancies
Source: PLoS One. 2026 Jul 29;21(7):e0354816. doi: 10.1371/journal.pone.0354816 (PMC13421757; doi:10.1371/journal.pone.0354816)
Supplement: Attachment — Submitted filename: Response to Reviewers.docx [file pone.0354816.s002.docx]

Response to the Editor and Reviewer

Manuscript ID: PONE-D-26-19382

Title: Longitudinal Changes in Laboratory Parameters and QTc During Isavuconazole Therapy in Japanese Patients with Hematologic Malignancies

Dear Editor and Reviewer,

We sincerely thank the Academic Editor and Reviewer #1 for their careful and constructive comments. We have revised the manuscript accordingly and have provided a point-by-point response below. All changes are indicated in the revised manuscript with tracked changes.

Reviewer #1, Comment 1:

In the Discussion section, please add comments on the limitations associated with the small size of the ATO subgroup and the lack of standardisation in the timing of ECG measurements, and briefly discuss how these factors may influence interpretation of the QTc findings.

Response:

We thank the reviewer for this important comment. We have expanded the Limitations section to more explicitly address both issues.

Changes in the manuscript: Discussion, Limitations section, pages 22–23, lines 332–350.

First, we clarified that the QTc analysis included only 17 patients receiving ATO-containing regimens and that the number of patients in the ATO-ISCZ group was limited. We now state that the estimated between-group QTc differences should be interpreted cautiously because they may be influenced by patient-level characteristics, treatment selection, and the distribution of repeated ECG measurements. We also clarify that mixed-effects modeling improved the use of available longitudinal data but could not fully overcome the limited sample size or establish causality.

Second, we further clarified that ECG measurements were performed according to clinical judgment rather than according to a prespecified schedule. We now note that differences in the timing and frequency of ECG assessments may have reflected the patients’ clinical condition, treatment phase, or perceived cardiovascular risk, thereby introducing potential surveillance bias and limiting direct comparability of QTc trajectories across groups. We added that prospective multicenter studies with larger cohorts and standardized ECG schedules are needed to confirm these findings.

Comment 2:

Consider adding a short explanation of the clinical significance of the observed QTc differences (e.g. 30–45 ms) and whether these differences are regarded as meaningful in routine practice. This would aid readers who are not specialists in this area.

Response:

We thank the reviewer for this valuable suggestion. We have added a paragraph to the Discussion section to clarify the clinical interpretation of the estimated QTc differences of approximately 30–45 ms.

Changes in the manuscript

Discussion section, pages 19-20, lines 294–305.

Although the estimated QTc differences were numerically substantial, their clinical implications should be interpreted cautiously. In routine clinical practice, QTc-related risk is assessed comprehensively on the basis of absolute QTc values, changes from baseline, concomitant risk factors, and the occurrence of arrhythmias, rather than QTc shortening itself. Therefore, the present findings should not be interpreted as evidence that QTc shortening per se represents a therapeutic benefit or that ISCZ prevents clinically significant arrhythmias.

Rather, the lower QTc values observed in the ATO-ISCZ group may suggest that concomitant ISCZ did not exacerbate ATO-associated QTc prolongation and may have contributed to maintaining lower QTc values during ATO therapy. However, because no clinically significant cardiac events occurred and this study was not powered to evaluate such outcomes, the effect of these QTc differences on arrhythmia risk remains uncertain.

Editorial Requirements

Editorial Requirement 1: PLOS ONE Style and File Naming

Response:

We reviewed the revised manuscript and submission files to ensure compliance with the PLOS ONE style requirements, including file naming and file designation at submission.

Editorial Requirement 2: Ethics Statement

Response:

We confirmed that the ethics statement appears only in the Methods section of the revised manuscript.

Editorial Requirement 3: Data Availability

We note that the data underlying the findings of this study contain potentially sensitive patient information and cannot be shared publicly because of ethical and legal restrictions related to patient confidentiality. If the data cannot be made publicly available for ethical or legal reasons, please explain the reasons on resubmission.

Response:

Thank you for this important clarification. We have revised our data-sharing approach and will provide the minimal de-identified dataset underlying the findings of this study as Supporting Information (S1 Data).

All direct identifiers and calendar dates were removed or replaced with study-specific relative time variables before public sharing. We have added a Data Availability Statement to the revised manuscript accordingly.

Changes in the manuscript:

Data Availability Statement, page 26, lines 405–407.

Editorial Requirement 4: Funding Information and Financial Disclosure

We note that the grant information provided in the “Funding Information” and “Financial Disclosure” sections did not match. Please ensure that the correct grant numbers are provided for the awards received for this study in the “Funding Information” section.

Response:

Thank you for bringing this discrepancy to our attention. We have revised the Funding Information and Financial Disclosure sections to ensure that they are fully consistent.

This study was conducted under a joint research agreement between Gifu University and Asahi Kasei Therapeutics Corporation. The study was supported through joint research funding provided to Gifu University under this agreement, with NK and AS designated as the responsible investigators. No grant number was assigned to this funding. The same funding information, including the absence of a grant number and the designation of NK and AS as the responsible investigators, has been entered consistently in both the Funding Information and Financial Disclosure sections of the revised submission.

We also clarified the role of the funder in the Financial Disclosure statement. Asahi Kasei Therapeutics Corporation contributed to study conceptualization through its employee co-authors, Mio Wada and Momoko Ohashi. The funder had no role in data collection, data management, statistical analysis, interpretation of the results, or the decision to publish. Mio Wada and Momoko Ohashi reviewed the manuscript and provided feedback; however, the original drafting and primary preparation of the manuscript were undertaken by the authors affiliated with Gifu University.

Editorial Requirement 5: Role of the Funder

Please state what role the funders took in the study. If the funders had no role, please state: “The funders had no role in study design, data collection and analysis, decision to publish, or preparation of the manuscript.” If this statement is not correct, please amend it as needed. Please include this amended Role of Funder statement in your cover letter.

Response:

Thank you for this request. We have clarified the role of the funder in the cover letter.

This study was conducted under a joint research agreement between Gifu University and Asahi Kasei Therapeutics Corporation. The funder contributed to study conceptualization through its employees and co-authors, Mio Wada and Momoko Ohashi. The funder had no role in data collection, data management, statistical analysis, interpretation of the results, or the decision to publish. Mio Wada and Momoko Ohashi reviewed the manuscript and provided feedback; however, the original drafting and primary preparation of the manuscript were undertaken by the authors affiliated with Gifu University.

Editorial Requirement 6: Abstract Consistency

Response:

Thank you for this reminder. We have revised the abstract in the online submission form to ensure that it is identical to the abstract in the revised manuscript.

Editorial Requirement 7: Reviewer-Suggested Citations

Response:

The reviewer did not recommend any specific publications for citation. Therefore, no additional citation was required in response to this item.

Editorial Requirement 8: Reference List

Response:

We reviewed the reference list to ensure that it is complete and accurate, including verification of the current status of cited publications.
