# Supplementary material for: Longitudinal changes in laboratory parameters and QTc during isavuconazole therapy in Japanese patients with hematologic malignancies
Source: PLoS One. 2026 Jul 29;21(7):e0354816. doi: 10.1371/journal.pone.0354816 (PMC13421757; doi:10.1371/journal.pone.0354816)
Supplement: Peer review history

# Peer review: Decision Letter 0

**DOI:** 10.1371/journal.pone.0354816.r001

**Authors:** Felix Bongomin

6 Jun 2026

PONE-D-26-19382

Longitudinal Changes in Laboratory Parameters and QTc During Isavuconazole Therapy in Japanese Patients with Hematologic Malignancies

PLOS One

Dear Dr. Kobayashi,

Thank you for submitting your manuscript to PLOS ONE. After careful consideration, we feel that it has merit but does not fully meet PLOS ONE’s publication criteria as it currently stands. Therefore, we invite you to submit a revised version of the manuscript that addresses the points raised during the review process.

Please submit your revised manuscript by Jul 21 2026 11:59PM. If you will need more time than this to complete your revisions, please reply to this message or contact the journal office at plosone@plos.org. When you're ready to submit your revision, log on to https://www.editorialmanager.com/pone/ and select the 'Submissions Needing Revision' folder to locate your manuscript file.

Please include the following items when submitting your revised manuscript:

- A letter that responds to each point raised by the academic editor and reviewer(s). You should upload this letter as a separate file labeled 'Response to Reviewers'.
- A marked-up copy of your manuscript that highlights changes made to the original version. You should upload this as a separate file labeled 'Revised Manuscript with Track Changes'.
- An unmarked version of your revised paper without tracked changes. You should upload this as a separate file labeled 'Manuscript'.

If you would like to make changes to your financial disclosure, please include your updated statement in your cover letter. Guidelines for resubmitting your figure files are available below the reviewer comments at the end of this letter.

If applicable, we recommend that you deposit your laboratory protocols in protocols.io to enhance the reproducibility of your results. Protocols.io assigns your protocol its own identifier (DOI) so that it can be cited independently in the future. For instructions see: https://journals.plos.org/plosone/s/submission-guidelines#loc-laboratory-protocols. Additionally, PLOS ONE offers an option for publishing peer-reviewed Lab Protocol articles, which describe protocols hosted on protocols.io. Read more information on sharing protocols at https://plos.org/protocols?utm_medium=editorial-email&utm_source=authorletters&utm_campaign=protocols.

As the corresponding author, your ORCID iD is verified in the submission system and will appear in the published article. PLOS supports the use of ORCID, and we encourage all coauthors to register for an ORCID iD and use it as well. Please encourage your coauthors to verify their ORCID iD within the submission system before final acceptance, as unverified ORCID iDs will not appear in the published article. *Only*  the individual author can complete the verification step; PLOS staff *cannot*  verify ORCID iDs on behalf of authors.

We look forward to receiving your revised manuscript.

Kind regards,

Felix Bongomin, MB ChB, Ph.D., FECMM

Academic Editor

PLOS One

Journal Requirements:

When submitting your revision, we need you to address these additional requirements.

1. Please ensure that your manuscript meets PLOS ONE's style requirements, including those for file naming. The PLOS ONE style templates can be found at

https://journals.plos.org/plosone/s/file?id=wjVg/PLOSOne_formatting_sample_main_body.pdf and https://journals.plos.org/plosone/s/file?id=ba62/PLOSOne_formatting_sample_title_authors_affiliations.pdf

2.Your ethics statement should only appear in the Methods section of your manuscript. If your ethics statement is written in any section besides the Methods, please delete it from any other section.

3. In the online submission form, you indicated that the data underlying the findings of this study contain potentially sensitive patient information and cannot be shared publicly because of ethical and legal restrictions related to patient confidentiality. The minimal anonymized dataset will be made available to qualified researchers upon reasonable request, subject to approval by the relevant institutional ethics committee and in accordance with institutional policies. Requests for data access may be directed to the Ethics Committee of the Graduate School of Medicine, Gifu University.

All PLOS journals now require all data underlying the findings described in their manuscript to be freely available to other researchers, either 1. In a public repository, 2. Within the manuscript itself, or 3. Uploaded as supplementary information.

This policy applies to all data except where public deposition would breach compliance with the protocol approved by your research ethics board. If your data cannot be made publicly available for ethical or legal reasons (e.g., public availability would compromise patient privacy), please explain your reasons on resubmission and your exemption request will be escalated for approval.

4. We note that the grant information you provided in the ‘Funding Information’ and ‘Financial Disclosure’ sections do not match.

When you resubmit, please ensure that you provide the correct grant numbers for the awards you received for your study in the ‘Funding Information’ section.

5. Thank you for stating the following financial disclosure:

This study was funded by Asahi Kasei Therapeutics Corporation  (URL:https://www.asahi-kasei.co.jp/pharma/en/) as joint research funding.

Nobuhiro Kanemura and Akio Suzuki has received research funding from Asahi Kasei Therapeutics Corporation.

Mio Wada and Momoko Ohashi are employees of Asahi Kasei Therapeutics Corporation.

Author contributions, Mio Wada: Conceptualization, Momoko Ohashi: Conceptualization

Please state what role the funders took in the study. If the funders had no role, please state: "The funders had no role in study design, data collection and analysis, decision to publish, or preparation of the manuscript."

If this statement is not correct you must amend it as needed.

Please include this amended Role of Funder statement in your cover letter; we will change the online submission form on your behalf.

6. Please amend either the abstract on the online submission form (via Edit Submission) or the abstract in the manuscript so that they are identical.

7. If the reviewer comments include a recommendation to cite specific previously published works, please review and evaluate these publications to determine whether they are relevant and should be cited. There is no requirement to cite these works unless the editor has indicated otherwise.

8. Please review your reference list to ensure that it is complete and correct. If you have cited papers that have been retracted, please include the rationale for doing so in the manuscript text, or remove these references and replace them with relevant current references. Any changes to the reference list should be mentioned in the rebuttal letter that accompanies your revised manuscript. If you need to cite a retracted article, indicate the article’s retracted status in the References list and also include a citation and full reference for the retraction notice.

[Note: HTML markup is below. Please do not edit.]

Reviewers' comments:

Reviewer's Responses to Questions

**Comments to the Author**

1. Is the manuscript technically sound, and do the data support the conclusions?

The manuscript must describe a technically sound piece of scientific research with data that supports the conclusions. Experiments must have been conducted rigorously, with appropriate controls, replication, and sample sizes. The conclusions must be drawn appropriately based on the data presented.

Reviewer #1: Yes

**********

2. Has the statistical analysis been performed appropriately and rigorously?

Reviewer #1: Yes

**********

3. Have the authors made all data underlying the findings in their manuscript fully available?

The PLOS Data policy requires authors to make all data underlying the findings described in their manuscript fully available without restriction, with rare exception (please refer to the Data Availability Statement in the manuscript PDF file). The data should be provided as part of the manuscript or its supporting information, or deposited to a public repository. For example, in addition to summary statistics, the data points behind means, medians and variance measures should be available. If there are restrictions on publicly sharing data—e.g. participant privacy or use of data from a third party—those must be specified.

Reviewer #1: Yes

**********

4. Is the manuscript presented in an intelligible fashion and written in standard English?

PLOS ONE does not copyedit accepted manuscripts, so the language in submitted articles must be clear, correct, and unambiguous. Any typographical or grammatical errors should be corrected at revision, so please note any specific errors here.

Reviewer #1: Yes

**********

5. Review Comments to the Author

Please use the space provided to explain your answers to the questions above. You may also include additional comments for the author, including concerns about dual publication, research ethics, or publication ethics. (Please upload your review as an attachment if it exceeds 20,000 characters)

Reviewer #1: Dear Authors,

As I am not an expert in electrophysiology or mixed-effects models, I focused this review on the study’s overall design, clarity, and clinical interpretability rather than on technical details. From a general clinical and methodological perspective, the manuscript is well written and logically structured. The study objectives are clearly stated, and the retrospective design and patient selection criteria are described transparently. The longitudinal analyses of laboratory values and QTc intervals are also presented in an easy-to-understand manner. The main findings-namely, that isavuconazole demonstrated a favourable safety profile in Japanese patients with haematological malignancies and appeared to be safe even during arsenic trioxide therapy-seem clinically plausible based on the data presented.

My suggestions are as follows;

1. In the Discussion section, please add comments on the limitations associated with the small size of the ATO subgroup and the lack of standardisation in the timing of ECG measurements, and briefly discuss how these factors may influence interpretation of the QTc findings.

2. Consider adding a short explanation of the clinical significance of the observed QTc differences (e.g. 30-45 ms) and whether these differences are regarded as meaningful in routine practice. This would aid readers who are not specialists in this area.

I have no further major concerns from a general clinical perspective.

**********

6. PLOS authors have the option to publish the peer review history of their article (what does this mean?). If published, this will include your full peer review and any attached files.

If you choose “no”, your identity will remain anonymous but your review may still be made public.

**Do you want your identity to be public for this peer review?**  For information about this choice, including consent withdrawal, please see our Privacy Policy.

Reviewer #1: **Yes:**  Takashi Kamio

**********

[NOTE: If reviewer comments were submitted as an attachment file, they will be attached to this email and accessible via the submission site. Please log into your account, locate the manuscript record, and check for the action link "View Attachments". If this link does not appear, there are no attachment files.]

To ensure your figures meet our technical requirements, please review our figure guidelines: https://journals.plos.org/plosone/s/figures

You may also use PLOS’s free figure tool, NAAS, to help you prepare publication quality figures: https://journals.plos.org/plosone/s/figures#loc-tools-for-figure-preparation.

NAAS will assess whether your figures meet our technical requirements by comparing each figure against our figure specifications.

# Author comment: Author response to Decision Letter 1

**DOI:** 10.1371/journal.pone.0354816.r002

9 Jul 2026

Response to the Editor and Reviewer

Manuscript ID: PONE-D-26-19382

Title: Longitudinal Changes in Laboratory Parameters and QTc During Isavuconazole Therapy in Japanese Patients with Hematologic Malignancies

Dear Editor and Reviewer,

We sincerely thank the Academic Editor and Reviewer #1 for their careful and constructive comments. We have revised the manuscript accordingly and have provided a point-by-point response below. All changes are indicated in the revised manuscript with tracked changes.

Reviewer #1, Comment 1:

In the Discussion section, please add comments on the limitations associated with the small size of the ATO subgroup and the lack of standardisation in the timing of ECG measurements, and briefly discuss how these factors may influence interpretation of the QTc findings.

Response:

We thank the reviewer for this important comment. We have expanded the Limitations section to more explicitly address both issues.

Changes in the manuscript: Discussion, Limitations section, pages 22–23, lines 332–350.

First, we clarified that the QTc analysis included only 17 patients receiving ATO-containing regimens and that the number of patients in the ATO-ISCZ group was limited. We now state that the estimated between-group QTc differences should be interpreted cautiously because they may be influenced by patient-level characteristics, treatment selection, and the distribution of repeated ECG measurements. We also clarify that mixed-effects modeling improved the use of available longitudinal data but could not fully overcome the limited sample size or establish causality.

Second, we further clarified that ECG measurements were performed according to clinical judgment rather than according to a prespecified schedule. We now note that differences in the timing and frequency of ECG assessments may have reflected the patients’ clinical condition, treatment phase, or perceived cardiovascular risk, thereby introducing potential surveillance bias and limiting direct comparability of QTc trajectories across groups. We added that prospective multicenter studies with larger cohorts and standardized ECG schedules are needed to confirm these findings.

Comment 2:

Consider adding a short explanation of the clinical significance of the observed QTc differences (e.g. 30–45 ms) and whether these differences are regarded as meaningful in routine practice. This would aid readers who are not specialists in this area.

Response:

We thank the reviewer for this valuable suggestion. We have added a paragraph to the Discussion section to clarify the clinical interpretation of the estimated QTc differences of approximately 30–45 ms.

Changes in the manuscript

Discussion section, pages 19-20, lines 294–305.

Although the estimated QTc differences were numerically substantial, their clinical implications should be interpreted cautiously. In routine clinical practice, QTc-related risk is assessed comprehensively on the basis of absolute QTc values, changes from baseline, concomitant risk factors, and the occurrence of arrhythmias, rather than QTc shortening itself. Therefore, the present findings should not be interpreted as evidence that QTc shortening per se represents a therapeutic benefit or that ISCZ prevents clinically significant arrhythmias.

Rather, the lower QTc values observed in the ATO-ISCZ group may suggest that concomitant ISCZ did not exacerbate ATO-associated QTc prolongation and may have contributed to maintaining lower QTc values during ATO therapy. However, because no clinically significant cardiac events occurred and this study was not powered to evaluate such outcomes, the effect of these QTc differences on arrhythmia risk remains uncertain.

Editorial Requirements

Editorial Requirement 1: PLOS ONE Style and File Naming

Response:

We reviewed the revised manuscript and submission files to ensure compliance with the PLOS ONE style requirements, including file naming and file designation at submission.

Editorial Requirement 2: Ethics Statement

Response:

We confirmed that the ethics statement appears only in the Methods section of the revised manuscript.

Editorial Requirement 3: Data Availability

We note that the data underlying the findings of this study contain potentially sensitive patient information and cannot be shared publicly because of ethical and legal restrictions related to patient confidentiality. If the data cannot be made publicly available for ethical or legal reasons, please explain the reasons on resubmission.

Response:

Thank you for this important clarification. We have revised our data-sharing approach and will provide the minimal de-identified dataset underlying the findings of this study as Supporting Information (S1 Data).

All direct identifiers and calendar dates were removed or replaced with study-specific relative time variables before public sharing. We have added a Data Availability Statement to the revised manuscript accordingly.

Changes in the manuscript:

Data Availability Statement, page 26, lines 405–407.

Editorial Requirement 4: Funding Information and Financial Disclosure

We note that the grant information provided in the “Funding Information” and “Financial Disclosure” sections did not match. Please ensure that the correct grant numbers are provided for the awards received for this study in the “Funding Information” section.

Response:

Thank you for bringing this discrepancy to our attention. We have revised the Funding Information and Financial Disclosure sections to ensure that they are fully consistent.

This study was supported by joint research funding from Asahi Kasei Therapeutics Corporation to Nobuhiro Kanemura and Akio Suzuki. No grant number was assigned to this funding. The same funding information, including the absence of a grant number, has been entered consistently in both the Funding Information and Financial Disclosure sections of the revised submission.

We also clarified the role of the funder in the Financial Disclosure statement. Asahi Kasei Therapeutics Corporation contributed to study conceptualization through its employee co-authors, Mio Wada and Momoko Ohashi. The funder had no role in data collection, data management, statistical analysis, interpretation of the results, or the decision to publish. Mio Wada and Momoko Ohashi reviewed the manuscript and provided feedback; however, the original drafting and primary preparation of the manuscript were undertaken by the authors affiliated with Gifu University.

Editorial Requirement 5: Role of the Funder

Please state what role the funders took in the study. If the funders had no role, please state: “The funders had no role in study design, data collection and analysis, decision to publish, or preparation of the manuscript.” If this statement is not correct, please amend it as needed. Please include this amended Role of Funder statement in your cover letter.

Response:

Thank you for this request. We have clarified the role of the funder in the cover letter.

This study was conducted under a joint research agreement between Gifu University and Asahi Kasei Therapeutics Corporation. The funder contributed to study conceptualization through its employees and co-authors, Mio Wada and Momoko Ohashi. The funder had no role in data collection, data management, statistical analysis, interpretation of the results, or the decision to publish. Mio Wada and Momoko Ohashi reviewed the manuscript and provided feedback; however, the original drafting and primary preparation of the manuscript were undertaken by the authors affiliated with Gifu University.

Editorial Requirement 6: Abstract Consistency

Response:

Thank you for this reminder. We have revised the abstract in the online submission form to ensure that it is identical to the abstract in the revised manuscript.

Editorial Requirement 7: Reviewer-Suggested Citations

Response:

The reviewer did not recommend any specific publications for citation. Therefore, no additional citation was required in response to this item.

Editorial Requirement 8: Reference List

Response:

We reviewed the reference list to ensure that it is complete and accurate, including verification of the current status of cited publications.

Attachment Submitted filename: Response to Reviewers.docx

# Peer review: Decision Letter 1

**DOI:** 10.1371/journal.pone.0354816.r003

**Authors:** Felix Bongomin

14 Jul 2026

Longitudinal Changes in Laboratory Parameters and QTc During Isavuconazole Therapy in Japanese Patients with Hematologic Malignancies

PONE-D-26-19382R1

Dear Dr. Kobayashi,

We’re pleased to inform you that your manuscript has been judged scientifically suitable for publication and will be formally accepted for publication once it meets all outstanding technical requirements.

Within one week, you’ll receive an e-mail detailing the required amendments. When these have been addressed, you’ll receive a formal acceptance letter and your manuscript will be scheduled for publication.

An invoice will be generated when your article is formally accepted. Please note, if your institution has a publishing partnership with PLOS and your article meets the relevant criteria, all or part of your publication costs will be covered. Please make sure your user information is up-to-date by logging into Editorial Manager at Editorial Manager® and clicking the ‘Update My Information' link at the top of the page. For questions related to billing, please contact billing support.

If your institution or institutions have a press office, please notify them about your upcoming paper to help maximize its impact. If they’ll be preparing press materials, please inform our press team as soon as possible -- no later than 48 hours after receiving the formal acceptance. Your manuscript will remain under strict press embargo until 2 pm Eastern Time on the date of publication. For more information, please contact onepress@plos.org.

Kind regards,

Felix Bongomin, MB ChB, Ph.D., FECMM

Academic Editor

PLOS One

Additional Editor Comments (optional):

Reviewers' comments:

Reviewer's Responses to Questions

**Comments to the Author**

1. If the authors have adequately addressed your comments raised in a previous round of review and you feel that this manuscript is now acceptable for publication, you may indicate that here to bypass the “Comments to the Author” section, enter your conflict of interest statement in the “Confidential to Editor” section, and submit your "Accept" recommendation.

Reviewer #1: All comments have been addressed

**********

2. Is the manuscript technically sound, and do the data support the conclusions?

The manuscript must describe a technically sound piece of scientific research with data that supports the conclusions. Experiments must have been conducted rigorously, with appropriate controls, replication, and sample sizes. The conclusions must be drawn appropriately based on the data presented.

Reviewer #1: Yes

**********

3. Has the statistical analysis been performed appropriately and rigorously?

Reviewer #1: Yes

**********

4. Have the authors made all data underlying the findings in their manuscript fully available?

The PLOS Data policy requires authors to make all data underlying the findings described in their manuscript fully available without restriction, with rare exception (please refer to the Data Availability Statement in the manuscript PDF file). The data should be provided as part of the manuscript or its supporting information, or deposited to a public repository. For example, in addition to summary statistics, the data points behind means, medians and variance measures should be available. If there are restrictions on publicly sharing data—e.g. participant privacy or use of data from a third party—those must be specified.

Reviewer #1: Yes

**********

5. Is the manuscript presented in an intelligible fashion and written in standard English?

PLOS ONE does not copyedit accepted manuscripts, so the language in submitted articles must be clear, correct, and unambiguous. Any typographical or grammatical errors should be corrected at revision, so please note any specific errors here.

Reviewer #1: Yes

**********

6. Review Comments to the Author

Please use the space provided to explain your answers to the questions above. You may also include additional comments for the author, including concerns about dual publication, research ethics, or publication ethics. (Please upload your review as an attachment if it exceeds 20,000 characters)

Reviewer #1: Thank you for your careful revision of the manuscript and your clear responses to my previous comments. The limitations related to the small ATO subgroup and the non-standardized timing of ECG measurements are now appropriately discussed, and the clinical interpretation of the observed QTc changes has been clarified. From a general clinical and methodological perspective, I do not have further major concerns, and I find the current version suitable to proceed in the editorial process.

**********

7. PLOS authors have the option to publish the peer review history of their article (what does this mean?). If published, this will include your full peer review and any attached files.

If you choose “no”, your identity will remain anonymous but your review may still be made public.

**Do you want your identity to be public for this peer review?**  For information about this choice, including consent withdrawal, please see our Privacy Policy.

Reviewer #1: No

**********

# Editor report: Acceptance letter

**DOI:** 10.1371/journal.pone.0354816.r004

**Authors:** Felix Bongomin

PONE-D-26-19382R1

PLOS One

Dear Dr. Kobayashi,

I'm pleased to inform you that your manuscript has been deemed suitable for publication in PLOS One. Congratulations! Your manuscript is now being handed over to our production team.

At this stage, our production department will prepare your paper for publication. This includes ensuring the following:

* All references, tables, and figures are properly cited

* All relevant supporting information is included in the manuscript submission,

* There are no issues that prevent the paper from being properly typeset

You will receive further instructions from the production team, including instructions on how to review your proof when it is ready. Please keep in mind that we are working through a large volume of accepted articles, so please give us a few days to review your paper and let you know the next and final steps.

Lastly, if your institution or institutions have a press office, please let them know about your upcoming paper now to help maximize its impact. If they'll be preparing press materials, please inform our press team within the next 48 hours. Your manuscript will remain under strict press embargo until 2 pm Eastern Time on the date of publication. For more information, please contact onepress@plos.org.

You will receive an invoice from PLOS for your publication fee after your manuscript has reached the completed accept phase. If you receive an email requesting payment before acceptance or for any other service, this may be a phishing scheme. Learn how to identify phishing emails and protect your accounts at https://explore.plos.org/phishing.

If we can help with anything else, please email us at customercare@plos.org.

Thank you for submitting your work to PLOS ONE and supporting open access.

Kind regards,

PLOS ONE Editorial Office Staff

on behalf of

Dr. Felix Bongomin

Academic Editor

PLOS One
